# Intermittent Theta Burst Stimulation Increases Natural Oscillatory Frequency in Ipsilesional Motor Cortex Post-Stroke: A Transcranial Magnetic Stimulation and Electroencephalography Study

**DOI:** 10.3389/fnagi.2022.818340

**Published:** 2022-02-07

**Authors:** Qian Ding, Songbin Chen, Jixiang Chen, Shunxi Zhang, Yuan Peng, Yujie Chen, Junhui Chen, Xiaotong Li, Kang Chen, Guiyuan Cai, Guangqing Xu, Yue Lan

**Affiliations:** ^1^Department of Rehabilitation Medicine, Guangzhou First People’s Hospital, South China University of Technology, Guangzhou, China; ^2^Department of Rehabilitation Medicine, Guangdong Provincial People’s Hospital, Guangdong Academy of Medical Sciences, Guangzhou, China

**Keywords:** intermittent theta burst stimulation, TMS-EEG, natural frequency, stroke rehabilitation, evoked oscillatory response

## Abstract

**Objective:**

Intermittent theta burst stimulation (iTBS) has been widely used as a neural modulation approach in stroke rehabilitation. Concurrent use of transcranial magnetic stimulation and electroencephalography (TMS-EEG) offers a chance to directly measure cortical reactivity and oscillatory dynamics and allows for investigating neural effects induced by iTBS in all stroke survivors including individuals without recordable MEPs. Here, we used TMS-EEG to investigate aftereffects of iTBS following stroke.

**Methods:**

We studied 22 stroke survivors (age: 65.2 ± 11.4 years; chronicity: 4.1 ± 3.5 months) with upper limb motor deficits. Upper-extremity component of Fugl-Meyer motor function assessment and action research arm test were used to measure motor function of stroke survivors. Stroke survivors were randomly divided into two groups receiving either Active or Sham iTBS applied over the ipsilesional primary motor cortex. TMS-EEG recordings were performed at baseline and immediately after Active or Sham iTBS. Time and time-frequency domain analyses were performed for quantifying TMS-evoked EEG responses.

**Results:**

At baseline, natural frequency was slower in the ipsilesional compared with the contralesional hemisphere (*P* = 0.006). Baseline natural frequency in the ipsilesional hemisphere was positively correlated with upper limb motor function following stroke (*P* = 0.007). After iTBS, natural frequency in the ipsilesional hemisphere was significantly increased (*P* < 0.001).

**Conclusions:**

This is the first study to investigate the acute neural adaptations after iTBS in stroke survivors using TMS-EEG. Our results revealed that natural frequency is altered following stroke which is related to motor impairments. iTBS increases natural frequency in the ipsilesional motor cortex in stroke survivors. Our findings implicate that iTBS holds the potential to normalize natural frequency in stroke survivors, which can be utilized in stroke rehabilitation.

## Introduction

Stroke is a debilitating acquired neurological injury and the leading cause of adult disability over the world (Lloyd-Jones et al., 2010). Upper limb motor deficits frequently occur following stroke, which negatively impact quality of life in stroke survivors (Tedesco Triccas et al., 2019). Motor impairment following stroke has been suggested to arise from disruptions of structural and functional integrity at both local and global scales (Bonkhoff et al., 2020). Intermittent theta burst stimulation (iTBS) is a specific form of repetitive transcranial magnetic stimulation (rTMS) that can effectively enhance cortical excitability and modulate oscillatory dynamics in both stimulated area and remote brain regions (Suppa et al., 2016; Ding et al., 2021c). iTBS has been considered as a promising approach for stroke rehabilitation (Corti et al., 2012; Suppa et al., 2016).

Neural effects of iTBS are typically investigated by motor evoked potentials (MEP), which are muscular responses elicited by single-pulse TMS (Talelli et al., 2007; Di Lazzaro et al., 2008; Ding et al., 2021b). However, this approach is not applicable to stroke survivors in whom MEPs are not elicitable. Furthermore, MEPs reflect cortical excitability in only motor cortex but not non-motor brain regions. Concurrent use of TMS and electroencephalography (EEG) (i.e., TMS-EEG) would overcome the drawbacks of MEP measurements, which offers a chance to simultaneously monitor cortical activity in both stimulated area and the interconnected cortical networks (Casula et al., 2021). Importantly, as TMS-EEG can directly measure cortical reactivity and oscillatory dynamics regardless of the integrity of corticospinal tracts (Borich et al., 2016), it allows for investigating neural effects induced by iTBS in stroke survivors without recordable MEPs (Pellicciari et al., 2018).

TMS-EEG signals can be analyzed in both time and time-frequency domains. TMS-evoked potential (TEP) is a complex waveform time-locked to the TMS pulse. TEPs reflect the direct activation of the cortical neurons at the stimulated brain regions (Pellicciari et al., 2018; Tremblay et al., 2019). In the time domain, TEPs can be quantified either by the amplitude and latency of peaks, or by the area under the curve of the rectified signals over the electrodes of interest [i.e., local mean field power (LMFP)] or over the entire surface of the head [i.e., global mean field power (GMFP)] (Tremblay et al., 2019). It has been suggested that LMFP and GMFP take both the width and the amplitude of TEPs into account and do not require an obvious main peak to present (Tremblay et al., 2019). As TEP main peaks are not always present in stroke survivors, LMFP and GMFP have the advantage of quantifying TEPs in stroke studies (Tscherpel et al., 2020). The acute adaptations in LMFP and GMFP after iTBS have been investigated in healthy adults, but results remain inconsistent in current literature (Gedankien et al., 2017; Bai et al., 2021; Ozdemir et al., 2021). Some studies reported no change in LMFP or GMFP after iTBS (Gedankien et al., 2017; Ozdemir et al., 2021), while one study reported reduced GMFP after iTBS (Bai et al., 2021). The adaptations in LMFP and GMFP after iTBS have not been investigated in stroke survivors.

Time-frequency domain analysis offers possibility to study the functional specificity of cortical oscillations in each frequency band [i.e., evoked oscillatory response (EOR)] (Thut and Miniussi, 2009). The adaptations in EOR after iTBS have been investigated in healthy adults, but results were inconsistent (Casula et al., 2016; Chung et al., 2017; Bai et al., 2021). One study reported a reduction in alpha band EOR after iTBS (Bai et al., 2021), while some studies reported an increase in theta (Chung et al., 2017) or beta (Casula et al., 2016) band EOR after iTBS. Besides EOR, time-frequency domain analysis also allows for the study of natural frequency (i.e., the frequency with the maximal power in a specific brain region) (Tremblay et al., 2019). Each brain area preserves its natural frequency of oscillations when stimulated with TMS (Casula et al., 2016). Natural frequency reflects the intrinsic dynamics of the corresponding thalamocortical circuits as well as the intrinsic GABA transmissions and provides important insights in the aftereffects of plasticity-inducing protocols on cortical activities (Rosanova et al., 2009; Tremblay et al., 2019; Ferrarelli and Phillips, 2021). It has been suggested that iTBS elevates the amount of ongoing activity in the connections between the stimulation site and its interconnected brain regions, including the thalamocortical pathway (Bestmann et al., 2004; Suppa et al., 2008). As thalamus is the structure that generates high-frequency oscillations, strengthened thalamocortical connections after iTBS could increase natural frequency in the stimulated brain region (Ferrarelli et al., 2012; Casula et al., 2016). To our knowledge, no study has investigated the adaptations of natural frequency after iTBS in either healthy adults or stroke survivors.

Following stroke, disruption of the intra- and inter- hemispheric network architecture may result in cortical deafferentation from subcortical structures, especially thalamus (Tscherpel et al., 2020). As thalamocortical neurons have been implicated in generating fast oscillations (Ferrarelli et al., 2012), impaired thalamocortical connections following stroke may contribute to a slowing of natural frequency in the ipsilesional hemisphere (IH) (Tscherpel et al., 2020). The alterations of natural frequency following stroke has been reported in a recent study (Tscherpel et al., 2020). Tscherpel et al. (2020) observed slowing of IH natural frequency following stroke as well as a positive correlation between natural frequency and motor function in stroke survivors. Based on limited number of studies, it remains unclear whether natural frequency could be used as a biomarker indicating stroke recovery.

In present study, we used concurrent TMS and EEG to investigate the aftereffects of iTBS in stroke survivors. We hypothesized that (1) natural frequency is slower in the ipsilesional compared with the contralesional hemisphere (CH); (2) there is a positive correlation between IH natural frequency and motor function; (3) IH natural frequency is increased after the application of iTBS. These results would have potential implications for understanding the influences of iTBS on cortical oscillatory dynamics in stroke survivors.

## Materials and Methods

### Subjects

Twenty-two stroke survivors were recruited into this study. Each subject provided written, informed consent prior to enrollment and participation. Approval for the experimental procedures was attained from Guangzhou First People’s Hospital Human Research Ethics Committee (reference number: K-2021-130-01). The study was carried out in conformity with standards set by the Declaration of Helsinki. Stroke survivors were included if they had a single stroke less than 18 months prior to enrollment. All participants were screened for eligibility to receive TMS and excluded if they were pregnant; using medications that reduce seizure threshold; or any metal or implanted devices that might be affected by TMS (Rossi et al., 2009). As substances like alcohol and caffeine can influence the aftereffects of iTBS, participants were asked not to consume alcohol or caffeine prior to the experiment (Chung et al., 2017). Stroke survivors were excluded from this study if they had cognitive impairment (i.e., the score of Montreal Cognitive Assessment was below 22/30) (Nasreddine et al., 2005) and cannot comprehend or follow three step commands (Ding et al., 2018). Nineteen out of twenty-two stroke survivors were free from lesions in the primary motor cortex (M1). Demographic characteristics are described in Tables 1, 2.

**TABLE 1:** Patients’ demographic and clinical characteristics.

	Age, years	Sex	Paretic side	Type of stroke	Months after stroke onset	UE FMA (0–66)	ARAT (0–57)
	
	Mean ± SD (range)	Male/ Female	Right /Left	Ischemic/ Hemorrhagic	Mean ± SD (range)	Mean ± SD (range)	Mean ± SD (range)
Active iTBS group (*n* = 11)	62.4 ± 14.0 (35–77)	9/2	5/6	9/2	6.0 ± 4.8 (1–18)	29.1 ± 18.0 (4–58)	28.7 ± 18.2 (0–54)
Sham iTBS group (*n* = 11)	64.4 ± 12.2 (42–79)	9/2	5/6	9/2	4.5 ± 4.1 (1–15)	34.9 ± 21.3 (4–66)	29 ± 22.9 (0–57)

*UE FMA refers to upper-extremity component of the Fugl-Meyer Motor Function Assessment, indicating motor impairments in stroke survivors (Fugl-Meyer et al., 1975).*

*ARAT refers to Action Research Arm Test, indicating upper extremity performance (i.e., coordination and dexterity) in neurological populations (Lang et al., 2006).*

*SD refers to standard deviation.*

*No significant difference in chronicity, UE FMA, or ARAT was revealed between subjects in Active and Sham iTBS groups.*

**TABLE 2:** Stroke characteristics.

Subject number	Sex	Age (years)	Paretic hand	Chronicity (months)	Type of stroke	Lesion location	UE FMA	RMT (MSO%)	iTBS stimulation intensity (MSO%)	iTBS stimulation intensity (RMT%)
S01	M	62	R	4	Ischemic	Basal ganglia	9	100	40	40
S02	M	72	L	2	Ischemic	Basal ganglia, periventricular white matter	43	80	40	50
S03	F	64	R	2	Ischemic	Frontal/temporal/ parietal lobe	4	100	40	40
S04	M	70	L	8	Ischemic	Pons	37	70	40	57
S05	M	57	R	8	Hemorrhagic	Frontal/parietal lobe	47	55	39	71
S06	M	77	L	1	Ischemic	Centrum semiovale, corona radiata, basal ganglia	58	70	40	57
S07	F	35	L	5	Ischemic	Frontal/parietal lobe	49	100	40	40
S08	M	69	R	11	Ischemic	Corona radiata	18	100	40	40
S09	M	70	R	3	Ischemic	Basal ganglia	26	75	40	53
S10	M	75	L	4	Ischemic	Pons	5	100	40	40
S11	M	35	L	18	Hemorrhagic	Basal ganglia	24	100	40	40
C01	M	78	L	3	Ischemic	Corona radiata, basal ganglia	60	70	40	57
C02	M	70	R	2	Ischemic	Basal ganglia	26	75	40	53
C03	M	42	L	6	Hemorrhagic	Pons	21	100	40	40
C04	F	79	R	2	Ischemic	Thalamus/ occipital lobe	36	40	28	70
C05	M	60	R	9	Ischemic	Frontal/parietal/ temporal lobe	4	100	40	40
C06	M	43	L	1	Ischemic	Frontal/parietal lobe, basal ganglia, corona radiata	64	20	14	70
C07	M	75	L	15	Ischemic	Parietal/temporal lobe	9	100	40	40
C08	F	58	R	1	Ischemic	Internal capsule	42	100	40	40
C09	M	68	L	3	Ischemic	Frontal/parietal/ temporal lobe, basil ganglia	66	30	21	70
C10	M	72	R	1	Hemorrhagic	Parietal/temporal lobe	44	40	28	70
C11	M	63	L	6	Ischemic	Basal ganglia, corona radiata, centrum semiovale, frontal lobe	12	100	40	40

*UE FMA refers to upper-extremity component of the Fugl-Meyer Motor Function Assessment.*

*M refers to male, and F refers to female.*

*L refers to left, and R refers to right.*

*S01–11 indicate stroke survivors in the Active iTBS group.*

*C01–11 indicate stroke survivors in the Sham iTBS group.*

*Stroke lesions of all subjects except S03, S07 and C05 were free from the primary motor cortex.*

### Experimental Procedure

This was a single-session, sham-controlled, randomized single-blinded study. Participants were randomly assigned to the experimental (Active iTBS) and control (Sham iTBS) groups, with eleven participants in each group. Participants were blinded with the group they were assigned to. Upper-extremity component of Fugl-Meyer motor function assessment (FMA) and action research arm test (ARAT) were used to assess motor impairment and upper limb motor function of stroke survivors, respectively. TMS-EEG recordings were performed at baseline and immediately after the completion of iTBS.

### Intermittent Theta Burst Stimulation

A NS5000 Magnetic Stimulator (YIRUIDE Medical Co., Wuhan, China) equipped with a 70 mm figure-of-eight coil was used for TMS delivery (biphasic pulses, pulse width = 350 ms). TBS involves the application of a burst of three pulses at 50 Hz repeated at 5 Hz. iTBS involves the application of a 2 s train of TBS repeated every 10 s for a total of 192 s (Huang et al., 2005).

Prior to the application of iTBS, resting motor threshold (RMT) determination was performed in both hands in a random order. Surface electromyography was recorded from the first dorsal interosseus (FDI). Participants were seated comfortably in a chair and were asked to keep their eyes open throughout the experiment (Chung et al., 2017; Ding et al., 2018). During TMS, the coil was positioned tangentially 45 degrees to the midline. Participants were instructed to keep their arms relaxed while determining the optimal scalp position for eliciting maximal responses in the FDI (Ding et al., 2018). RMT was determined as the minimum intensity required to evoke 5 out of 10 MEPs greater than 50 μV at rest (Chen et al., 1998). A neuronavigation system (Visor2, ANT Neuro, Hengelo, Netherlands) was used to ensure consistent coil positioning over the optimal scalp position (i.e., motor hotspot) throughout the experiment (Ding et al., 2021a,c). For the individuals in whom MEPs in the IH cannot be elicited even with 100% MSO (*n* = 6 in the Active iTBS group and *n* = 5 in the Sham iTBS group), RMT in the IH was considered as 100% maximum stimulator output (MSO).

iTBS was applied over the motor hotspot in the ipsilesional M1. The iTBS stimulation intensity was set at 70% RMT in the IH (Volz et al., 2016; Ding et al., 2021c). During the application of iTBS, participants were instructed to remain static. As 40% MSO is the upper limit for iTBS with the NS5000 Magnetic Stimulator, iTBS stimulation intensity was set at 40% MSO if the calculated stimulation intensity was greater than 40% MSO (Ding et al., 2021c). For sham stimulation, the same stimulation intensity was used as for iTBS. The TMS coil was held perpendicular to the skull, touching the skull with the rim opposite the handle during sham stimulation (Nettekoven et al., 2014).

### Transcranial Magnetic Stimulation-Electroencephalography Recordings

During TMS-EEG recording, participants were seated comfortably in a sound-shielded, dimly lit room. TMS was performed using a NS5000 Magnetic Stimulator (YIRUIDE Medical Co., Wuhan, China) (biphasic pulses, pulse width = 350 ms). A neuronavigation system (Visor2, ANT Neuro, Hengelo, Netherlands) was used during the application of TMS (Ding et al., 2021a).

TMS-evoked EEG responses were recorded using a TMS-compatible EEG cap (ANT Neuro, Enschede, Netherlands) with 64 Ag/AgCl electrodes in a layout based on the extended international 10–20 system for electrodes placement (Jurcak et al., 2007; Tamburro et al., 2020). All channels were referenced online to CPz and amplified with an eego amplifier (ANT Neuro, Enschede, Netherlands). Data were sampled at 8,000 Hz with impedances kept below 5 kΩ for all channels throughout data collection. To prevent EEG auditory evoked potentials and eye muscle reactions induced by the TMS click, participants wore inserted earplugs during TMS-EEG recordings (Ter Braack et al., 2015; Tscherpel et al., 2020). To minimize bone conduction produced by TMS, we placed a thin layer of plastic film between the TMS coil and the EEG cap during testing (Massimini et al., 2007; Tscherpel et al., 2020).

TMS-EEG testing was performed in both hemispheres in a random order. During TMS-EEG recordings, 50 TMS pulses were applied on M1 (i.e., motor hotspot) in each hemisphere with a 5–8 s interval between two adjacent stimuli. Stimulation intensity was set at 80% RMT. On one hand, this intensity is suggested to be above the threshold of a significant EEG response (Tremblay et al., 2019; Tscherpel et al., 2020). On the other hand, as this intensity is below RMT, muscular responses are unlikely to be induced, which limits reafferent somatosensory feedback that is known to influence on the EEG responses (Tremblay et al., 2019). Of note, for the individuals in whom MEPs in the IH were not elicitable, we used RMT in the CH as a reference for setting stimulation intensities in the IH and defined ipsilesional motor hotspot based on anatomical landmarks (i.e., the hand knob) (Tscherpel et al., 2020).

### Data Analysis

#### Transcranial Magnetic Stimulation-Electroencephalography Analysis

Acquired EEG signals were analyzed off-line using MATLAB2019b (Mathworks, Inc., Natick, MA, United States) with customized scripts. EEGLAB toolbox (version 14.1.2b) (Delorme and Makeig, 2004) and TMSEEG toolbox (Atluri et al., 2016) were used for data preprocessing. Continuous EEG data were epoched around the test TMS pulse (−1,000 to + 1,000 ms). Each data trial was baseline corrected with the mean of the pre-stimulus period from −700 to −200 ms (Atluri et al., 2016; Sun et al., 2018). Data from −5 to 20 ms was discarded to eliminate the large TMS artifact. Trials and channels that were outliers with high-frequency power were labeled and removed by guided visual inspection (Sun et al., 2018) (number of artifact-free trials: Active iTBS group: 44.3 ± 4.5, Sham iTBS group: 44.1 ± 2.2). TMS decay artifacts were cleaned by removing characteristic noise components extracted with independent component analysis (ICA) (number of ICA components removed: Active iTBS group: 4.9 ± 1.1, Sham iTBS group: 4.8 ± 0.8). The signals were then band-pass filtered to 1–45 Hz with a notch filter (50 Hz). After the signals were filtered, a second round of ICA was performed to eliminate other noise components (e.g., ocular, cardiac or muscular artifacts, etc.) (Delorme et al., 2007) (number of ICA components removed: Active iTBS group: 2.5 ± 1.4, Sham iTBS group: 2.7 ± 0.8). Any missing channels were linearly interpolated (number of interpolated channels: Active iTBS group: 5.2 ± 1.3, Sham iTBS group: 5.2 ± 1.6). All channels were then referenced to the average across all electrodes and sampled down to 1,000 Hz.

After preprocessing, EEG data trials were averaged for each recording condition to obtain TEPs. When visualizing the TEPs, we found that electrodes near the stimulating coil suffered from residual artifacts for up to + 30 ms, which cannot be completely cleaned by ICA (Komssi et al., 2004; Rogasch and Fitzgerald, 2013). To be consistent with previous studies (Rogasch and Fitzgerald, 2013; Gordon et al., 2018), data analysis in current study focused on TEPs after + 30 ms from the TMS onset. LMFP was computed as the square root of squared TEPs averaged across the five channels surrounding the stimulated motor cortex (Left motor cortex: C1, C3, FC1, FC3, Cz; Right motor cortex: C2, C4, FC2, FC4, Cz) (Tscherpel et al., 2020). To minimize the effect of possible artifacts occurring at the time of stimulation, LMFP was calculated over a 30–200 ms time window with using the following formula:


(1) $$LMFP\left(t\right)=\sqrt{\frac{\left[\sum_{i}^{k}{\left(V_{i}\left(t\right)-V_{mean}\left(t\right)\right)}^{2}\right]}{K}}$$

where *t* is time, *K* is the number of channels, *V*_*i*_ is the voltage in channel *i* averaged across subjects, and *V*_*mean*_ is the averaged voltage in the channels of interest (Casula et al., 2016).

GMFP was computed as the square root of squared TEPs averaged across all active channels on the entire surface of the head. GMFP was calculated over a 30–200 ms time window with using the following formula:


(2) $$GMFP\left(t\right)=\sqrt{\frac{\left[\sum_{i}^{k}{\left(V_{i}\left(t\right)-V_{mean}\left(t\right)\right)}^{2}\right]}{K}}$$

where *t* is time, *K* is the number of channels, *V*_*i*_ is the voltage in channel *i* averaged across subjects, and *V*_*mean*_ is the averaged voltages in all active channels (Lehmann and Skrandies, 1980; Pellicciari et al., 2018).

Spectral features in the time-frequency domain were evaluated by computing the event-related spectral perturbation (ERSP) based on Morlet wavelet transform as follows:


(3) $$ERSP\left(f,t\right)=\frac{1}{n}\sum_{k=1}^{n}{\left|F_{k}\left(f,t\right)\right|}^{2}$$

where for *n* trials, the spectral estimate *F* was computed at trial *k*, at frequency *f* and time *t* (Casula et al., 2018; Koch et al., 2020).

EOR was computed by averaging the oscillatory activity of channels surrounding the stimulated motor cortex (Left motor cortex: C1, C3, FC1, FC3, Cz; Right motor cortex: C2, C4, FC2, FC4, Cz). To minimize the effect of TMS artifacts, the frequency values were calculated by averaging the EOR values over a 30–200 ms time window. Subsequently, the spectral power in the frequency ranges between 2–4 Hz (delta), 4–8 Hz (theta), 8–13 Hz (alpha), and 13–30 Hz (beta) was extracted from the wavelet dataset (Pellicciari et al., 2018). Natural frequency was then calculated as the frequency with the largest cumulated ERSP upon stimulated motor cortex between 5–50 Hz (Tremblay et al., 2019; Tscherpel et al., 2020).

#### Statistical Analysis

Statistical analyses were performed in JMP Pro Version 13.2 (SAS Institute Inc., Cary, NC, United States) and FieldTrip toolbox (Oostenveld et al., 2011) in MATLAB2019b. Non-parametric cluster-based permutation tests by means of the Monte Carlo method were conducted to assess differential changes in LMFP and GMFP after iTBS between groups in each hemisphere. Comparisons were first made across time points for each iTBS condition (within-comparison). Between-group comparisons were performed using change-from-baseline score (i.e., after iTBS–before iTBS) (Chung et al., 2019). Monte Carlo *P*-values were calculated on 3,000 random permutations, and t-values within every cluster were summed up for cluster-level statistics in each permutation. The proportion of random permutations with larger test statistics than the observed one was the significance probability. Time points were considered significant when > 10 successive *t*-tests reached the significance threshold (*P* < 0.025) (Casula et al., 2016).

Linear mixed effects (LME) modeling was performed to test differential changes in EOR and natural frequency after iTBS between groups in each hemisphere. Group, Timepoint, and Group × Timepoint interaction were included as fixed effects, and subject was included as a random effect. Timepoint was set as repeated covariance structure. Normality of the residuals was visually assessed for each model with conditional residual quantile-quantile plots, and all were found to reasonably conform to the assumption of normality. *Post hoc* tests were performed when F-tests were significant. Multiple comparisons between Timepoints or Groups were performed with Tukey–Kramer adjustment.

The normality of data was tested using the Kolmogorov-Smirnov test before conducting correlation analyses. For the data that met the normality assumption, Pearson correlations were performed to investigate the relationship between baseline and changes in neurophysiological measures (e.g., LMFP, GMFP, EOR and natural frequency) and subject characteristics (e.g., age, chronicity, FMA, and ARAT). For the data that violated the normality assumption, Spearman correlations were performed instead. For all analyses, statistical significance was established at *P* < 0.05. *Post hoc* power analyses were performed using G*power (Version 3.1) (Faul et al., 2007) and JMP pro (Version 13.2) to determine whether current study had enough power to detect the differences between conditions, which calculated the power (1–β) as a function of α (0.05), the sample size, and the population effect size (Cohen, 1988; Chung et al., 2019).

## Results

**FIGURE 1:**
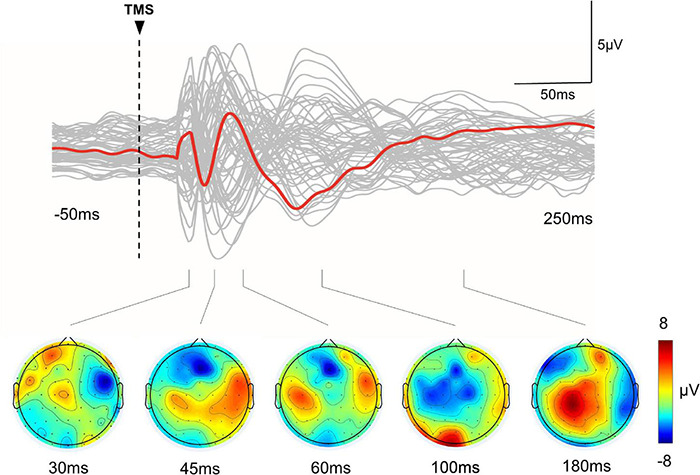
TMS-evoked EEG potentials in a representative subject. Top row: The gray curves represent TEP in each channel, and the red bold curves represent the averaged TEP in the channels surrounding the stimulated motor cortex (C4). Bottom row: Topographic plots of the TMS-evoked responses at 30, 45, 60, 100, and 180 ms post-TMS.

All participants tolerated iTBS well and no adverse events were reported. TEPs in a representative patient are presented in Figure 1.

### Local Mean Field Power and Global Mean Field Power

**FIGURE 2:**
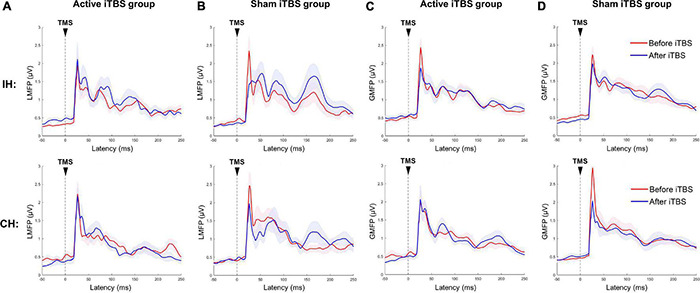
Local and global mean field power changes after iTBS. Data presented are group mean ± standard error. The top panel presents the data in the IH, and the bottom panel presents the data in the CH. **(A,B)** LMFP in the Active and Sham TBS group, respectively. **(C,D)** GMFP in the Active and Sham TBS group, respectively. The red curves represent LMFP or GMFP before iTBS, and the blue curves represent LMFP or GMFP after iTBS. There was no significant change in LMFP or GMFP after iTBS in either group.

The cluster-based permutation test did not reveal any significant change after Active or Sham iTBS in either hemisphere (*P*’s > 0.05) (Figure 2).

### Transcranial Magnetic Stimulation-Evoked Oscillatory Response

**FIGURE 3:**
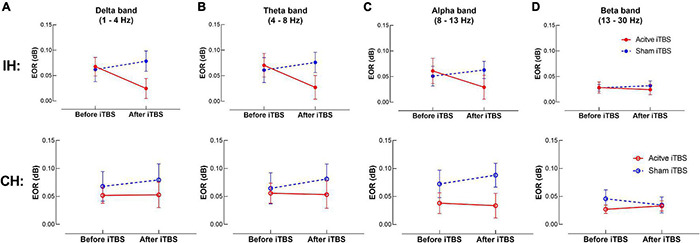
TMS-evoked oscillatory response (EOR) changes after iTBS. Data presented are group mean ± standard error. The top panel presents the data in the IH, and the bottom panel presents the data in the CH. **(A–D)** presents EOR before and after iTBS in the delta, theta, alpha and beta bands, respectively. The red circles represent the Active iTBS group, and the blue circles represent the Sham iTBS group. There was no significant change in EOR after iTBS in any frequency band in either group.

The LME modeling did not reveal any significant main effect or interaction in any frequency band in either hemisphere (*P*’s > 0.05) (Figure 3).

### Natural Frequency

**FIGURE 4:**
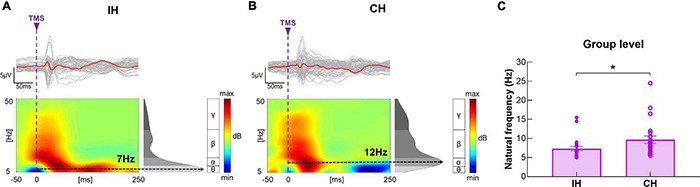
Differences in baseline natural frequency between hemispheres. **(A,B)** Illustration of TEP, ERSP and natural frequency in the IH and CH in a representative subject. The gray curves represent TEP in each channel, and the red bold curves represent the averaged TEP in the channels surrounding the stimulated motor cortex. The ERSP plots show the TMS-evoked oscillatory responses in amplitude and duration, with black dotted lines highlighting the frequency with the highest power (i.e., natural frequency). **(C)** Data presented are group mean ± standard error. Baseline natural frequency was significantly slower in the IH compared with CH in the entire sample. The solid circles represent the IH, and the empty circles represent the CH.

At baseline, natural frequency in the IH was significantly slower compared with the CH (*P* = 0.006, power = 0.67) (Figure 4).

**FIGURE 5:**
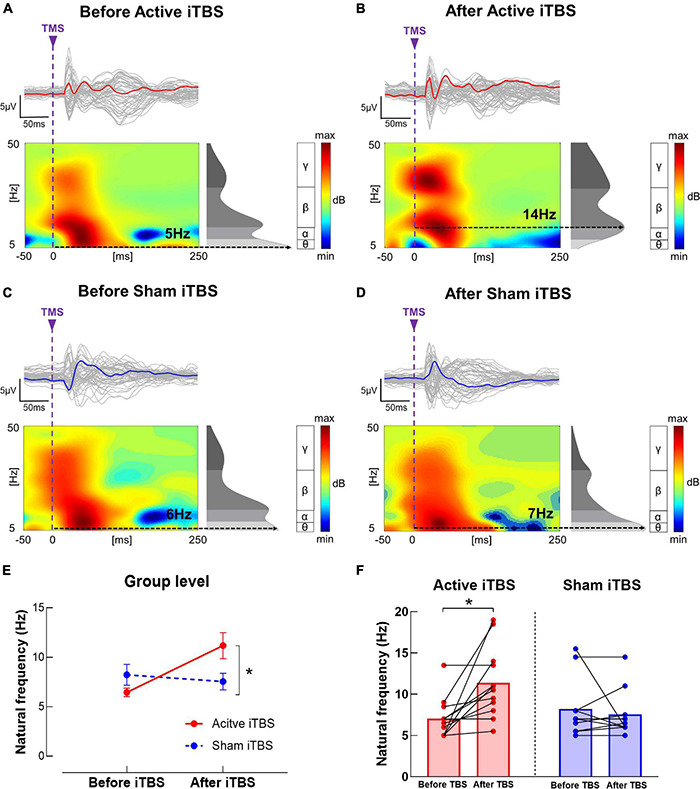
Natural frequency changes after iTBS. **(A–D)** Illustration of TEP, ERSP and natural frequency in the IH before and after iTBS in representative subjects in the Active **(A,B)** and Sham **(C,D)** iTBS groups. The gray curves represent TEP in each channel, and the bold curves represent the averaged TEP in the channels surrounding the stimulated motor cortex. The ERSP plots show the TMS-evoked oscillatory responses in amplitude and duration, with black dotted lines highlighting the frequency with the highest power (i.e., natural frequency). **(E)** Data presented are group mean ± standard error. In the Active iTBS group, IH natural frequency was significantly increased after iTBS; while in the Sham iTBS group, there was no significant change in natural frequency after iTBS. **(F)** Illustration of individual changes in IH natural frequency after iTBS in both groups. The red circles represent the Active iTBS group, and the blue circles represent the Sham iTBS group.

In the IH, the LME modeling revealed significant main effect of Timepoint and Timepoint × Group interaction [*F*_(1, 20)_ = 6.00, *P* = 0.024, power = 0.58; *F*_(1, 20)_ = 10.73, *P* = 0.004, power = 0.81, respectively] in natural frequency. *Post hoc* revealed that in the Active iTBS group, natural frequency was significantly increased after iTBS (*P* < 0.001), while in the Sham iTBS group, natural frequency was not significantly changed after iTBS (*P* > 0.05) (Figure 5).

In the CH, the LME modeling did not reveal any significant main effect or interaction in natural frequency (*P*’s > 0.05).

### Correlation Analysis

**FIGURE 6:**
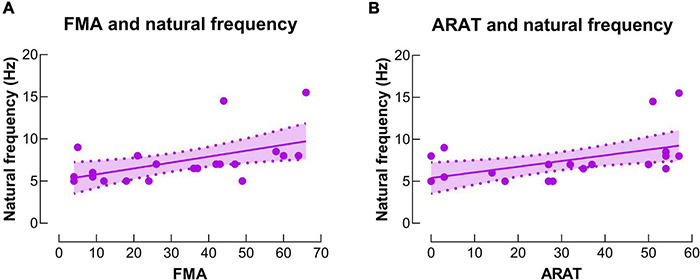
Correlations between natural frequency and motor function. **(A)** There was a significant positive correlation between natural frequency in the IH and upper extremity Fugl-Meyer assessment (FMA) score. **(B)** There was a significant positive correlation between natural frequency in the IH and action research arm test (ARAT) score.

Significant positive correlations were observed between baseline IH natural frequency and motor function (including ARAT and FMA) (r’s = 0.56 and 0.54, *P*’s = 0.007 and 0.009, power’s = 0.81 and 0.77, respectively) (Figure 6). No significant correlation was observed between other neurophysiological measures (i.e., LMFP, GMFP and EOR) and subject characteristics (i.e., age, chronicity and motor function) (*P*’s > 0.05).

## Discussion

In this study, we conducted concurrent TMS and EEG measurements at baseline and immediately after iTBS in stroke survivors. To our knowledge, this is the first study that used TMS-EEG to investigate the aftereffects of iTBS following stroke. At baseline, natural frequency was slower in the IH compared with CH, and IH natural frequency was positively correlated with upper limb motor function in stroke survivors. We also observed a significant increase in IH natural frequency after iTBS.

### Alterations in Natural Frequency Following Stroke

At baseline, natural frequency was significantly slower in the IH compare with CH in stroke survivors. The difference in natural frequency between hemispheres has not been previously investigated following stroke. This is the first study to report an imbalance in natural frequency between hemispheres post-stroke. Similar results have been reported in a recent study (Tscherpel et al., 2020), in which natural frequency was slower in the IH of stroke survivors compared with healthy adults. Natural frequency in the motor cortex has been widely investigated in healthy adults (Rosanova et al., 2009; Ferrarelli et al., 2012; Casula et al., 2016; Tscherpel et al., 2020). In most studies with healthy adults, motor cortex natural frequency falls in the beta band (i.e., 13–30 Hz) (Rosanova et al., 2009; Ferrarelli et al., 2012; Casula et al., 2016; Tscherpel et al., 2020), which appears to be faster than those observed in stroke survivors in current study (7 Hz and 10 Hz in the IH and CH, respectively). As there was no healthy control group at baseline comparison in current study, our results suggest that natural frequency tends to be slower in the IH relative to CH following stroke, but whether natural frequency in both hemispheres (especially CH) is slower in stroke survivors compared with healthy adults needs to be tested in future studies.

We observed a positive correlation between IH natural frequency and paretic hand motor function, that is, the more severely impaired stroke survivors tend to have slower natural frequency in the IH. The same correlation was also reported by Tscherpel et al. (2020). Apart from this cross-sectional correlation, Tscherpel et al. (2020) also reported positive correlations between improvements in the paretic arm motor function and changes in natural frequency during the course of stroke recovery, suggesting that motor recovery following stroke is accompanied by an increase in IH natural frequency. Together, natural frequency provides valuable insights in investigating neural mechanisms underlying motor deficits following stroke and has the potential to be used as a biomarker indicating stroke recovery.

The mechanisms underlying the alterations in natural frequency following stroke remains unclear. One possible mechanism is associated with stroke-related disruption of thalamocortical connections (Tscherpel et al., 2020). Functional magnetic resonance imaging studies have reported substantial disturbance of the intra- and inter- hemispheric network architecture following stroke (Grefkes et al., 2008; Carter et al., 2010), and these alterations have been suggested to be related to motor deficits in stroke survivors (Grefkes et al., 2008; Carter et al., 2010). Lesion-induced cortical deafferentation was observed from subcortical structures, especially thalamus (Tscherpel et al., 2020). As thalamocortical neurons have been implicated in generating fast oscillations (Ferrarelli et al., 2012), disconnection of thalamocortical pathway following stroke may result in reduced IH natural frequency.

Apart from structural disconnections, intrinsic deficits of cortical neurons following stroke could be another mechanism. GABAergic interneurons have been suggested to produce and sustain complex large-scale network oscillations in fast frequency bands (Benes and Berretta, 2001), which are important for integration and synchronous communication between brain regions and are implicated in many brain functions, including fine motor control (Varela et al., 2001; Sohn and Hallett, 2004; Canali et al., 2017). GABA-mediated intracortical inhibition has been reported to be reduced in the IH following stroke (Liepert et al., 2000; Swayne et al., 2008; Ding et al., 2018). Although reduction in GABA-mediated intracortical inhibition may elevate neural plasticity and promote motor recovery in the early stage post-stroke (Clarkson et al., 2010; Huynh et al., 2013), prolonged deficiency in GABAergic activity may hamper stroke recovery over a wider time span (Liepert, 2006; Marconi et al., 2011; Ding et al., 2018). As natural frequency has been suggested to reflect intrinsic GABA transmissions (Ferrarelli et al., 2012; Tremblay et al., 2019; Ferrarelli and Phillips, 2021), deficient GABA-mediated inhibitory processes could be associated with slowing of natural frequency following stroke, especially in the individuals with severe motor impairments.

### Effects of iTBS on Transcranial Magnetic Stimulation-Evoked Electroencephalography Responses

Transcranial magnetic stimulation-evoked EEG responses were quantified in the time and time-frequency domain in this study. In the time domain, we did not observe any significant change in LMFP or GMFP after iTBS. Several studies have investigated acute adaptations in LMFP and GMFP after iTBS applied on M1 in healthy adults, but the results lack a common thread (Gedankien et al., 2017; Bai et al., 2021; Ozdemir et al., 2021). No significant change in LMFP or GMFP after iTBS was observed in some studies (Gedankien et al., 2017; Ozdemir et al., 2021), but a reduction in GMFP after iTBS was reported in a recent study (Bai et al., 2021). Ozdemir et al. (2021) reported low reproducibility of iTBS-induced modulation of cortical responses (i.e., LMFP and GMFP) across two visits. The authors suggested that iTBS-induced neuromodulation may not be accurately reflected by TMS-EEG time domain parameters, possibly contributing to the inconsistent results in current literature (Ozdemir et al., 2021). Further studies are still needed to investigate whether TMS-EEG time domain parameters are good indicators for iTBS-induced neuromodulation.

In the time-frequency domain, we observed an increase in IH natural frequency after iTBS in stroke survivors. To our knowledge, the adaptation in natural frequency following TBS or rTMS has not been previously investigated in either healthy adults or stroke survivors, although natural frequency has been suggested to provide important insights in the aftereffects of plasticity-inducing protocols on cortical activities (Rosanova et al., 2009; Tremblay et al., 2019; Ferrarelli and Phillips, 2021). The mechanisms underlying the increase in natural frequency after iTBS are still unclear. As neural oscillations in cortical networks originated from interactions between cortical regions and the thalamus, natural frequency in a specific cortical region is thought to reflect the intrinsic dynamic of the corresponding thalamocortical circuits (Casula et al., 2016). It has been suggested that iTBS increases the amount of ongoing activity in the connections between the stimulation site and its interconnected brain regions, including the thalamocortical pathway (Bestmann et al., 2004; Suppa et al., 2008). As thalamus is the structure that generates high-frequency oscillations, strengthened thalamocortical connections after iTBS could increase natural frequency in the stimulated motor cortex (Ferrarelli et al., 2012; Casula et al., 2016).

We did not observe a significant change in EOR after iTBS in any frequency band. To our knowledge, adaptations in EOR after iTBS have only been investigated in healthy adults (Casula et al., 2016; Chung et al., 2017; Bai et al., 2021), but not in stroke survivors. Bai et al. (2021) applied iTBS on M1 and reported a reduction in alpha band EOR after iTBS. Chung et al. (Chung et al., 2017) and Casula et al. (2016) applied iTBS on non-motor areas (i.e., prefrontal cortex, cerebellum) and reported an increase in EOR in theta or beta band, respectively. Different stimulation sites of iTBS may account for inconsistent results among studies, as different brain regions preserve distinct oscillation properties (Rosanova et al., 2009; Tremblay et al., 2019). Different study populations [i.e., stroke survivors in current study *vs.* healthy adults (Bai et al., 2021)] and different methodological details (i.e., stimulation intensity of 80% RMT in current study *vs.* 110% RMT (Bai et al., 2021) for TMS-EEG recordings) may account for the inconsistent results between ours and Bai et al.’s (Bai et al., 2021) study. Based on the limited number of studies that investigated the adaptations in EOR after iTBS, further studies are needed to investigate how EOR is modulated after iTBS.

### Clinical Implications

This is the first study that used concurrent TMS-EEG to investigate the aftereffects of iTBS following stroke. Aftereffects of iTBS are typically investigated by muscle responses induced by single-pulse TMS, which is not applicable to stroke survivors without a measurable MEP (Byblow et al., 2015; Pellicciari et al., 2018). At this regard, TMS-EEG does not rely on the integrity of the corticospinal tract or other efferent and afferent pathways; instead, it directly assesses cortical reactivity and oscillatory dynamics (Borich et al., 2016; Tscherpel et al., 2020). Therefore, TMS-EEG allows for a standardized assessment post-stroke and holds the potential to provide novel biomarkers indicating neuroplastic changes in response to intervention.

As to quantifying TMS-evoked EEG responses, we included natural frequency, a novel TMS-EEG parameter that reflects the intrinsic dynamics of the corresponding thalamocortical circuits as well as the intrinsic GABA transmissions. In line with a recent study (Tscherpel et al., 2020), we observed slowing of natural frequency in the IH as well as a positive correlation between IH natural frequency and motor function in stroke survivors, suggesting that alterations in natural frequency might be associated with motor deficits post-stroke, and natural frequency could be used as a biomarker indicating stroke recovery. Furthermore, IH natural frequency was increased after iTBS, indicating that natural frequency can be normalized by iTBS in stroke survivors and iTBS has the potential to promote motor recovery following stroke.

### Limitations

We acknowledge limitations of present study. As a pilot study, the sample size of this study is small (*N* = 22). The chronicity of stroke survivors who participated in this study were within 18 months, so our findings may not be generalized to more chronic stroke survivors. Furthermore, most subjects in our sample (16 out of 22) were in the subacute phase of stroke (i.e., 1–6 months from stroke onset), which did not allow us to perform subgroup analysis for chronicity in current study. In future work, our results need to be tested in stroke survivors with a wider range of chronicity with larger sample sizes and to perform subgroup analysis for individuals in acute, subacute and chronic phases of stroke.

Auditory and somatosensory potentials arising from the TMS pulses would contaminate the direct cortical response to TMS and confound its interpretation, so strategies are needed to minimize those unwanted stimulation. In current study, we used ear plugs and plastic film under the coil to dampen the auditory and somatosensory stimulation (Massimini et al., 2007; Ter Braack et al., 2015; Tscherpel et al., 2020), but some recent studies suggested that auditory noise masking and foam padding could be more effective in attenuating auditory and somatosensory potentials (Conde et al., 2019; Rocchi et al., 2021). We acknowledge that the lack of using noise masking and foam padding is a limitation of current study. Further TMS-EEG studies are needed to apply noise masking and foam padding to minimize the auditory and somatosensory potentials caused by TMS pulses.

For sham iTBS condition, we used regular TMS coil that is tilted with an edge touching the head. This sham TBS approach produces a clicking sound that is very similar to an active TMS pulse, which has been widely adopted in previous TMS studies (Koch et al., 2009, 2020; Li et al., 2014; Nettekoven et al., 2014; Casula et al., 2016). However, the somatosensory perception produced by this sham TMS approach may not be as strong as active TMS, which possibly affects the arousal level of subjects in the two groups differently. Further studies are needed to use sham TMS coil with better mimic of somatosensory perception produced by active TMS.

Current study delivered only 50 pulses in each hemisphere when testing TMS-EEG (∼44 artifact-free trials), which is a small number of trials compared with many previous studies (Rosanova et al., 2009; Pellicciari et al., 2018; Tscherpel et al., 2020). As TMS-EEG testing was repeated four times in a single session for each subject (i.e., IH and CH, before and after iTBS), it was difficult for many stroke survivors to maintain static for such a long time. Therefore, we did not deliver more TMS pulses for TMS-EEG testing. As suggested in previous studies, adequate number of trials are critical for generating reliable TEPs (Rogasch and Fitzgerald, 2013; Tremblay et al., 2019). We acknowledge that the small number of trials is a limitation in current study which may negatively affect the reliability of current study. Cautions are needed when interpreting our results, and further studies with larger number of trials are needed to be conducted.

Present study took TMS-EEG measurements only at baseline and immediately after iTBS without a follow-up. We acknowledge that it would be more meaningful to take TMS-EEG measurements at multiple time points after iTBS, but it has already been a long experiment for stroke survivors, and many subjects could not tolerate for a longer time of data collection. Further studies are needed to measure TMS-EEG at multiple time points after iTBS.

### Conclusion

This is the first study to use concurrent TMS and EEG to investigate the aftereffects of iTBS in following stroke. We observed alterations in natural frequency following stroke which is related to motor impairments. Our results also provide evidence that iTBS increases natural frequency in stroke survivors. Our findings implicate that iTBS has the potential to normalize natural frequency in stroke survivors, which can be utilized in stroke rehabilitation.

## Data Availability Statement

The raw data supporting the conclusions of this article will be made available by the authors, without undue reservation.

## Ethics Statement

The studies involving human participants were reviewed and approved by Guangzhou First People’s Hospital Human Research Ethics Committee. The patients/participants provided their written informed consent to participate in this study. Written informed consent was obtained from the individual(s) for the publication of any potentially identifiable images or data included in this article.

## Author Contributions

YL, GX, and QD designed the experiments and wrote the manuscript. JiC and YP recruited the participants. QD, SC, JiC, SZ, XL, JuC, YC, and KC conducted the experiments. QD, SC, GC, and JiC reduced and analyzed the data. QD and YL interpreted the data. All authors contributed to the article and approved the submitted version.

## Conflict of Interest

The authors declare that the research was conducted in the absence of any commercial or financial relationships that could be construed as a potential conflict of interest.

## Publisher’s Note

All claims expressed in this article are solely those of the authors and do not necessarily represent those of their affiliated organizations, or those of the publisher, the editors and the reviewers. Any product that may be evaluated in this article, or claim that may be made by its manufacturer, is not guaranteed or endorsed by the publisher.

## Abbreviations

iTBS: intermittent theta burst stimulation
rTMS: repetitive transcranial magnetic stimulation
MEP: motor evoked potential
EEG: electroencephalography
TEP: TMS-evoked potential
LMFP: local mean field power
GMFP: global mean field power
EOR: evoked oscillatory response
IH: ipsilesional hemisphere
CH: contralesional hemisphere
FMA: Fugl-Meyer assessment
ARAT: action research arm test
RMT: resting motor threshold
FDI: first dorsal interosseus
M1: primary motor cortex
MSO: maximum stimulator output
ICA: independent component analysis
ERSP: event-related spectral perturbation
LME: linear mixed effects.
